# The impact of fast vs. slow rubidium-82 infusion profile on precision and accuracy of PET myocardial blood flow perfusion metrics using a 1-tissue compartment model

**DOI:** 10.1093/ehjimp/qyaf132

**Published:** 2025-10-24

**Authors:** Adrienne Koos, Richard V Milani, Cruz Velasco-Gonzalez, Daniel P Morin, Robert M Bober

**Affiliations:** Department of Internal Medicine, Ochsner Health, 1514 Jefferson Hwy, Jefferson, LA 70121, USA; Sutter Health, Center for Healthcare Innovation, San Francisco, CA, USA; Ochsner Center for Outcomes Research, Ochsner Health, New Orleans, LA, USA; Department of Cardiovascular Diseases, John Ochsner Heart and Vascular Institute, New Orleans, LA, USA; Queensland University School of Medicine, Ochsner Clinical School, New Orleans, LA, USA; Division of Electrophysiology, University of California, San Francisco, CA, USA; Sutter Health, Center for Healthcare Innovation, San Francisco, CA, USA; Department of Cardiovascular Diseases, John Ochsner Heart and Vascular Institute, New Orleans, LA, USA; Queensland University School of Medicine, Ochsner Clinical School, New Orleans, LA, USA; Sutter East Bay Medical Group, Sutter Health, San Francisco, CA, USA

**Keywords:** Rb-82 infusion profile, 3D positron emission tomography (PET), image quality

## Abstract

**Aims:**

Gould’s simplified retention model (GSRM), as implemented in the HeartSee software, demonstrates 10% same-day test-retest precision for PET-CT myocardial perfusion using a 50 or 20 mL/min Rb-82 infusions. HeartSee-GSRM also accurately quantifies resting myocardial blood flow (rMBF) in transmural scar (0.26 mL/min/g), aligning with reference standards. However, the impact for varying infusion rates on precision and accuracy of a 1-tissue compartment model (1-TCM) as implemented within 4DM software remains unclear. We assessed whether varying infusion rates of Rb-82 impacts 1-TCM precision and accuracy.

**Methods and results:**

Ninety-eight volunteers (Normals, Clinicals, and Infarcts), underwent 3D PET-CT stress testing. Three resting scans and two stress scans were performed with randomized fast (F) 50 mL/min or slow (S) 20 mL/min Rb-82 infusions. rMBF and stress MBF (sMBF) were calculated using 4DM software (1-TCM). Repeatability coefficients (RC) and coefficients of variance (COV) were calculated. Accuracy was assessed by comparing rMBF in infarcted myocardium (from 1-TCM and GSRM) against established reference standard for transmural myocardial scar (TMS). Fast infusion yielded better precision. RC was lower for F-F vs. S-S resting pairs (24.3% vs. 32.9%), and COV was lower (12.9% vs. 17.4%, *P* = 0.03). No difference in rMBF or sMBF was found between infusion rates (rMBF: 0.93 vs. 0.94 mL/min/g; sMBF: 2.23 vs. 2.30 mL/min/g). HeartSee GSRM produced rMBF values consistent with TMS (<0.30 mL/min/g), while 4DM 1-TCM overestimated rMBF (Fast: 0.79 ± 0.33; Slow: 0.82 ± 0.27 mL/min/g, *P* = 0.791), for both infusion profiles.

**Conclusion:**

Fast infusion improves 4DM 1-TCM precision, but 4DM 1-TCM overestimates rMBF in TMS regardless of infusion rate. HeartSee GSRM remains accurate and precise across profiles.

## Graphical Abstract

**Graphical Abstract:**
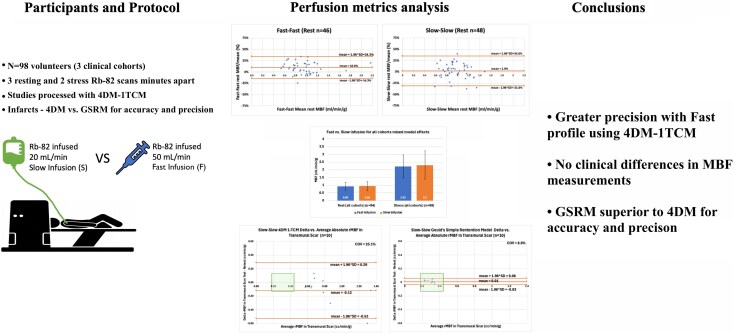

## Introduction

Positron emission tomography (PET) quantification of absolute MBF plays a crucial role in managing coronary artery disease (CAD). Recent studies have demonstrated that PET with MBF-guided decision-making in selecting patients for revascularization significantly improves post-revascularization MBF and reduces the rates of both death and myocardial infarction.^1–5^ Quantitative myocardial blood flow (MBF) with positron emission tomography (PET) has significant clinical potential, but its broad application requires standardization across technologies, techniques, kinetic models, and software. Both precision and accuracy must be optimized to ensure consistent clinical value.

Historically, PET was most widely applied in oncology FDG imaging, highlighting metabolic ‘hot spots’ in relatively stationary tissues at low injected activity. By contrast, cardiac PET with rubidium-82 (Rb-82) is ‘cold-spot’ perfusion imaging of a dynamic organ subject to contraction and respiratory motion, requiring high activity injections because of the tracer’s short half-life and the need to capture rapid first-pass kinetics.

Scanner hardware defined the initial limitations. Legacy 2D bismuth germanate (BGO) PET scanners provided stable count-rate performance for high-activity tracers, with manageable dead time, randoms, and scatter, but had limited spatial resolution, sensitivity, and image quality compared with later systems. As the PET industry—driven largely by oncology FDG applications—migrated to 3D acquisition with BGO and early LSO detectors, resolution and image quality improved, but quantification of high count-rate tracers such as Rb-82 became more challenging because computational power was insufficient to fully correct for dead time, scatter, and randoms. To address these challenges, constant-activity (slow) infusion profiles were introduced to reduce instantaneous detector load. With the adoption of 3D LSO scanners and, subsequently, digital PET systems, high-activity bolus dosing can be performed with accurate corrections.

Renaud et al. characterized the dynamic range of modern 3D PET systems for quantitative MBF imaging and demonstrated that accurate MBF values can be obtained across diverse scanners when the injected activity is optimized to remain below count-rate saturation thresholds.^6^ Importantly, the study showed that the dynamic performance of the various 3D camera systems differed substantially, with notable variability in count-rate capability and dead-time behaviour among vendors and models. These findings support the broader theme that standardization of PET MBF quantification across imaging systems remains a work in progress.

Kinetic modelling approaches evolved alongside these technical changes. The one-tissue compartment model (1-TCM) was introduced as a practical dynamic approach to describe Rb-82 kinetics under clinical imaging conditions. Lortie et al. demonstrated that a 1-compartment model provided excellent fits to human Rb-82 time–activity curves, with MBF estimates that correlated well with those obtained using 13N-ammonia.^7^ This practical implementation supported its adoption into commercial software packages.

In parallel, what is now referred to as Gould’s simplified retention model (GSRM) was developed through systematic experimental testing of multiple acquisition and modelling strategies. Several alternatives were directly compared, and the retention-based approach that remains in use today was determined experimentally to be the most practical and reproducible for Rb-82 bolus injections.^8,9^ This approach has several advantageous features. It does not depend on the detailed shape of the arterial input curve and reduces spill-over by acquiring separate arterial and myocardial static frames. Because myocardial uptake is measured over a longer time window, it has lower statistical noise than time–activity curve (TAC) fitting and is less affected by patient, cardiac, or respiratory motion. It uses an explicit partial-volume correction factor and has been shown to maintain reproducibility in the low-flow range where TAC methods are often most variable.^10^

Software implementations introduced further variability. Different models and corrections embedded in commercial packages (e.g. 4DM, HeartSee) have produced discordant MBF values from identical datasets. Accordingly, methodological factors—including infusion profiles—have been tested to assess precision across implementations. To date, there remains disagreement on whether constant-activity (slow) or fast infusion is preferable. In our prior work using GSRM-HeartSee on a contemporary 3D LSO scanner, we observed no significant difference in precision between slow and fast infusion profiles, but that study did not include 1-TCM. Published data with 1-TCM in 4DM have primarily focused on the slow/CA profile, and there is little information on same-day precision with fast infusion.

Standardization remains the overarching need. Current guidelines emphasize that each laboratory define its own ischaemic thresholds, reflecting the lack of consensus benchmarks for accuracy. A physiologic anchor is transmural scar (TMS), where resting MBF has been studied extensively since the 1970s using multiple tracers (Rb-82, 13N-ammonia, 15O-water), microspheres, kinetic models, and experimental species (humans, dogs, pigs). Across these diverse approaches, results consistently converge on a resting flow in TMS of approximately 0.30 mL/min/g or lower.^11^ This convergence represents a physiologic boundary condition that provides an objective reference for assessing MBF accuracy.

Accordingly, the present study aims to:

1. Determine whether the Rb-82 infusion rate (fast vs. slow) influences the precision of perfusion metrics using 1-TCM in 4DM.
2. Compare the precision of 1-TCM (4DM) vs. GSRM (HeartSee) applied to the same population with identical design and statistics.
3. Assess whether infusion profile influences the accuracy of MBF estimates in 1-TCM (4DM) and GSRM (HeartSee), using TMS as a physiologic reference.

## Materials and methods

The study was approved by the Ochsner Health Institutional Review Board and was registered at ClinicalTrials.gov (https://clinicaltrials.gov/ct2/show/NCT05286593). This study was performed in line with the principles of the Declaration of Helsinki. Informed consent was obtained from each participant. This study is a secondary analysis of data acquired in our previously published prospective study evaluating the impact of Rb-82 infusion profiles on test-retest precision of myocardial blood flow (MBF) using GSRM.^12^ In that study, 98 volunteers were enrolled and categorized into three cohorts [as defined in Supplementary data online, *Table S2*]:

1. Healthy, young, normal volunteers without cardiovascular disease or CAD risk factors.
2. Clinical patients with cardiovascular risk factors or stable coronary disease, and
3. Patients with prior transmural myocardial infarctions (TMS) confirmed by PET imaging.

**Figure 1:**
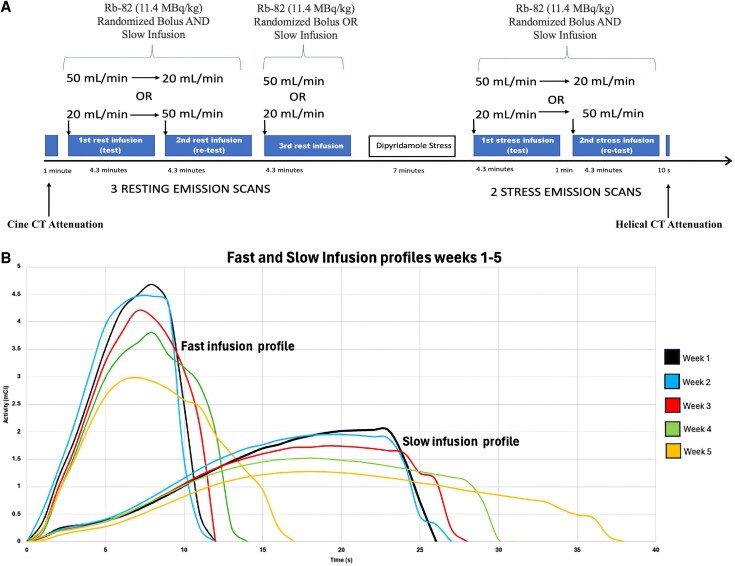
Patient protocol and Rubidium-82 infusion profiles. *Note.* Reprinted from Bober et al, 2024, J Nucl Cardiol. *A*) Subjects received three consecutive resting and two consecutive stress scans using two distinct Rb-82 infusion profiles performed 5 min apart and randomized as depicted. *B*) Weekly fast and slow infusion profiles over a 5-week period.

All participants underwent same-day, repeated rest-stress Rb-82 PET imaging with randomized fast (50 mL/min) and slow (20 mL/min) infusion profiles, as outlined in *Figure 1*. Study design, subject screening, image acquisition, and reconstruction methods were identical to the original protocol.

To address Objective 1, all 98 datasets (acquired using Fast and Slow infusion profiles) were re-analysed using the 1-TCM with 4DM software to evaluate the impact of the infusion profile on precision metrics of MBF. To address Objective 2, MBF values and precision metrics from the 1-TCM (analysed under both Fast and Slow infusion profiles) were compared directly to those previously obtained with GSRM in HeartSee, following the same study design and statistical methods. To address Objective 3, regions of transmural scar from each of the 20 subjects were used as a reference standard to assess the accuracy of rMBF estimates obtained with the 1-TCM and GSRM-HeartSee models under both infusion profiles. Accuracy metrics were compared across models and infusion conditions.

### Myocardial blood flow using 1-tissue compartment model

Datasets were systematically processed using the software package 4DM V2018.0.0.226 (INVIA, Ann Arbor, Michigan) between 10-2023 and 7-2024. The 4DM software uses a 1-tissue ‘Lortie’ compartment kinetic model (1-TCM) that has been described previously (9, 10). The arterial input is derived from time activity curves from a rectangular ROI at the mitral valve plane which can be adjusted to minimize spill-over from LV myocardium. Operator (AK), processed all studies *de novo*. Although 4DM features automatic motion correction, additional manual adjustments to the contours and motion correction of the dynamic sequences were performed as needed.

Each resting and stress MBF measurement was corrected for the patient-specific rate pressure product (RPP_cor_) using the equation: RPP_cor_ = rMBF/RPP x RPP_avg_. However, for the final analyses, no RPP-based adjustment of the rest or stress MBF values was required, as discussed in the Results section.

### Myocardial blood flow using a simple retention model

In the original analysis using GSRM (Bober et al, 2024)^12^, MBF was quantified using a simplified retention model implemented in HeartSee software (University of Texas Houston, Houston, TX). The arterial input function was derived from an early image frame corresponding to blood pool activity, and myocardial uptake was calculated from late summed frames. Partial volume corrections are automatically applied within the model and were determined experimentally with a one-dimensional tree phantom to be 0.85.

### Test re-test precision and repeatability

Two distinct metrics are commonly used to assess variability and precision of test-retest MBF data: 1) repeatability coefficient (RC), and 2) coefficient of variance (COV).^13,14^

The RC is the maximum difference that is likely to occur (i.e. in 95% of cases) between repeated measurements made **under the same conditions**. In the case of two repeated measurements, RC is calculated as 1.96 times the standard deviation (SD) of differences between the two measurements and can be visualized as the upper and lower bounds on Bland-Altman plots.^15,16^

The coefficient of variation (COV), a measure of dispersion, is calculated as the ratio of the SD of the differences between values to the mean of the measurements. In the case of two repeated measurements within a subject, COV is calculated by the equation:


$$COV=\frac{Standard\;deviation\;of\;the\;differences}{Mean\;of\;the\;measurements}\times 100\;$$

The higher the COV, the greater the level of dispersion around the mean. RC requires **identical conditions** (i.e. F-F or S-S pairings), whereas COV does not require identical conditions.^15^ Therefore, for resting conditions, both RC and COV are reported. At stress, subjects did not receive identical profiles and therefore only COV is reported.

### Transmural scar segment inclusion

TMS was defined as a continuous defect with ≥ 20% of the LV with relative uptake ≤50% without evidence of viability. Viability was determined either by previous PET-FDG, cardiac magnetic resonance imaging, or on clinical grounds (e.g. akinesis on echocardiography and Q-waves on electrocardiography; multiple prior noninvasive perfusion scans demonstrating a large, severe, fixed defect; or a history of ST-elevation myocardial infarction with late or no reperfusion). All patients in the Infarct group exhibited obvious, large regions of TMS with near absence of radiotracer uptake. However, not all segments with apparent defects were included in the analysis, due in part to the anatomical limitations of the standard 17-segment model, which does not always align precisely with coronary anatomy or physiology. To ensure consistent and physiologically meaningful inclusion, only segments that met the following criteria were selected: (1) the segments were adjacent to one another, forming a contiguous defect, (2) more than 50% of the segment was involved, (3) the summed rest score of the segment was ≥2, and (4) the segmental per cent radiotracer uptake was less than 65%. These criteria were based on prior studies and were designed to capture only the most severely affected regions for use as an internal reference standard.

### Statistical methods

Analyses were conducted using SAS/STAT 15.2 (SAS Institute) and Wizard 2.0.13 (Karelia Software). Results are reported as mean ± SD or median (IQR), as appropriate. Normality was assessed via Q-Q plots and Shapiro-Wilk, Kolmogorov-Smirnov, and Jarque-Bera tests (see Supplementary data online, *Table S1*, Supplementary data online, *Figures S1*–S2). Test-retest agreement was evaluated with Bland-Altman plots, intraclass correlation coefficient (ICC), and linear regression. Repeatability and variance were quantified using RC and COV, respectively; COVs for Fast and Slow infusions were compared using the Forkman test. Resting Fast–Fast vs. Slow–Slow test-retest differences were assessed via F-test. Primary comparisons of MBF between infusion profiles used a mixed model for crossover design (Infusion, Period as fixed effects; no carry-over effect: *P* = 0.1243 rest, *P* = 0.1849 stress). Secondary analysis assessed MBF differences across clinical groups and sexes; nonsignificant interactions (*P* > 0.05) were sequentially removed. Sensitivity and regression analyses evaluated generator age effects on MBF.

## Results

Ninety-eight volunteers (43 Normals, 34 Clinicals, and 21 Infarcts) completed the protocol consisting of 3 resting scans and 2 stress scans. Complete datasets from 3 subjects were excluded due to 1) abnormal venous anatomy, 2) malfunctioning IV, or 3) abnormal extracardiac uptake of Rb-82 which degraded myocardial images. One resting dataset was excluded for an aborted IV injection. Six stress datasets were excluded for 1) detectable levels of caffeine, 2) vasovagal response during stress, or 3) camera malfunction. Thus, there were a total of 94 resting data sets and 89 stress datasets in the final analysis. The patients’ demographic data are described in Supplementary data online, *Table S2*.

### Haemodynamics during rest and stress

Haemodynamic measurements are summarized in Supplementary data online, *Table S3*. Repeated measures ANOVA produced no significant interaction between infusion profiles for any of the measurements at each condition.

During sequential testing, the diastolic BP decreased. There was a statistically significant decrease in rDBP (mmHg) between Rest1 and Rest2 (64.9 ± 15.0 vs. 62.5 ± 14.8, *P* = 0.012) and between Rest1 and Rest 3 (64.9 ± 15.0 vs. 62.1 ± 13.7, *P* = 0.003), as well as between Rest2 and Rest3 (*P* = 0.0218). However, resting rate pressure product (RPP) did not demonstrate variation between infusion profiles. Across all three resting scans, paired t-testing showed no statistical difference (*P* = 0.932). Test–retest analysis likewise demonstrated no significant difference for resting RPP (*P* = 0.711), or for stress (*P* = 0.829).

RPP test-retest correlation was high for both rest and stress RPP (R = 0.94, *P* < 0.001; and R = 0.85, *P* < 0.001), as demonstrated in Supplementary data online, *Figure S3*. Test-retest changes in MBF were not correlated with changes in RPP, as shown in Supplementary data online, *Figure S3*.^12,13^ Therefore, in the final analyses no RPP-based adjustment of the rest or stress MBF values was performed.

### Test-retest precision and infusion profile in a 1-TCM-4DM

**Figure 2:**
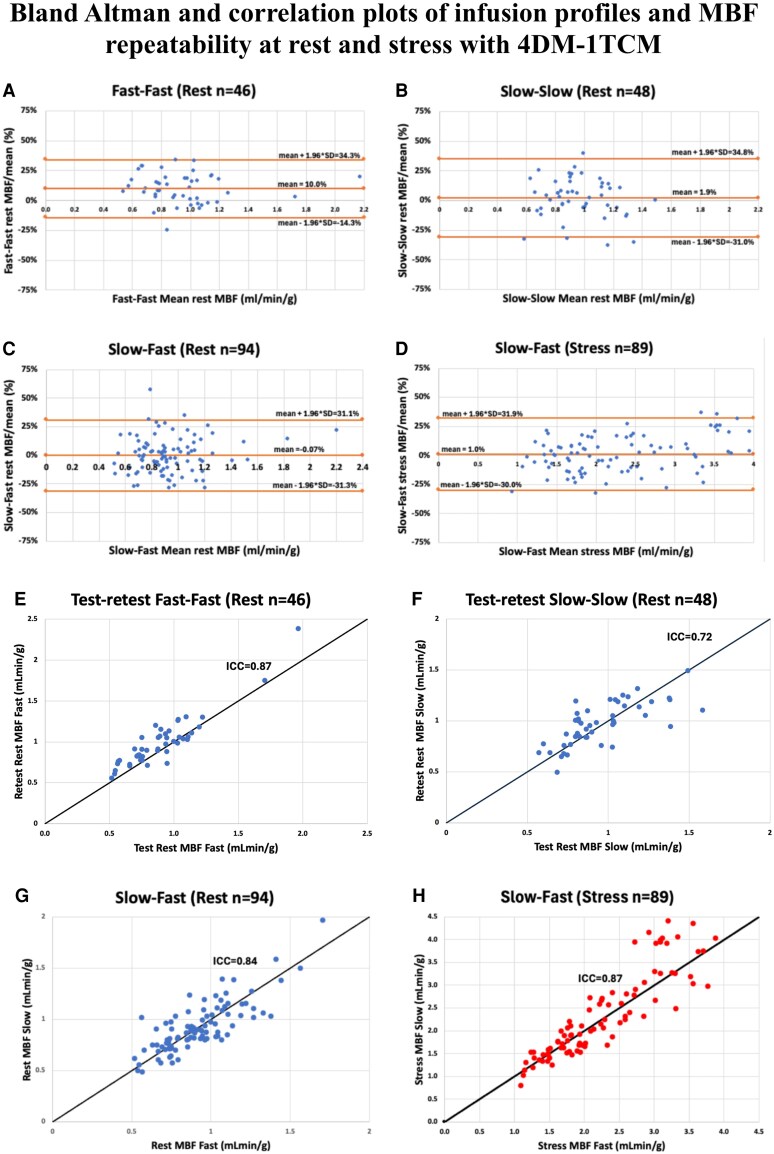
Bland Altman and correlation plots of infusion profiles and MBF repeatability at rest and stress.

All data were normally distributed as depicted in Supplementary data online, *Figures S1* and *S2* and Supplementary data online, *Table S1*. Bland-Altman (test-retest per cent differences) and scatter plots for test-retest pairs are shown in *Figure 2*. Bland-Altman plots of absolute differences are shown in Supplementary data online, *Figure S4*.

Test-retest precision metrics are summarized in *Table 1*. The F-F infusion pairs demonstrated greater precision than S-S pairs (i.e. lower COV and RC). To facilitate comparison across protocols and models, consistent with prior literature, RC was also expressed as a percentage of the mean MBF value (RC%), calculated as: **RC%**  **=**  **100**  **×**  **(1.96**  **×**  **SD of differences)/Mean MBF**. The RC% for F-F pairs and S-S pairs was 24.3% and 32.9%, respectively (*Table 1*). At rest, the COV for F-F pairs and S-S pairs was 12.9% and 17.4%, respectively (*P* = 0.03). At stress, COV for F-S pairs was 9.6%. ICC for rMBF ranged between 0.72-0.87 and for stress was 0.87.

**Table 1:** Test re-test precision

	Rest Fast-Fast (n = 46)	Rest Slow-Slow (n = 48)	*P*-values Fast vs. Slow	Stress Fast-Slow (n = 89)
Standard Deviation of Test-retest% (absolute)	12.4% (0.12)	16.8% (0.17)	**0**.**04** **0.03**	15.8% (0.42)
Mean Difference of Test—Retest% (absolute)	10.0% (0.09)	1.94% (0.01)	**0.02**	1.00% (0.07)
RC = 1.96*SD of test-retest% (absolute)	24.3% (0.18)	32.9% (0.40)	N/A	N/A
Mean Test and retest (mL/min/g)	0.94	0.96	0.714	1.98
COV = absolute SD differences/mean	12.9%	17.4%	**0.03** ^a^	9.6%
ICC	0.87	0.72	N/A	0.87

COV coefficient of variation, RC repeatability coefficient, ICC intraclass correlation coefficient.

^a^Forkman Test.

### Comparison of F and S profiles on myocardial blood flow

**Figure 3:**
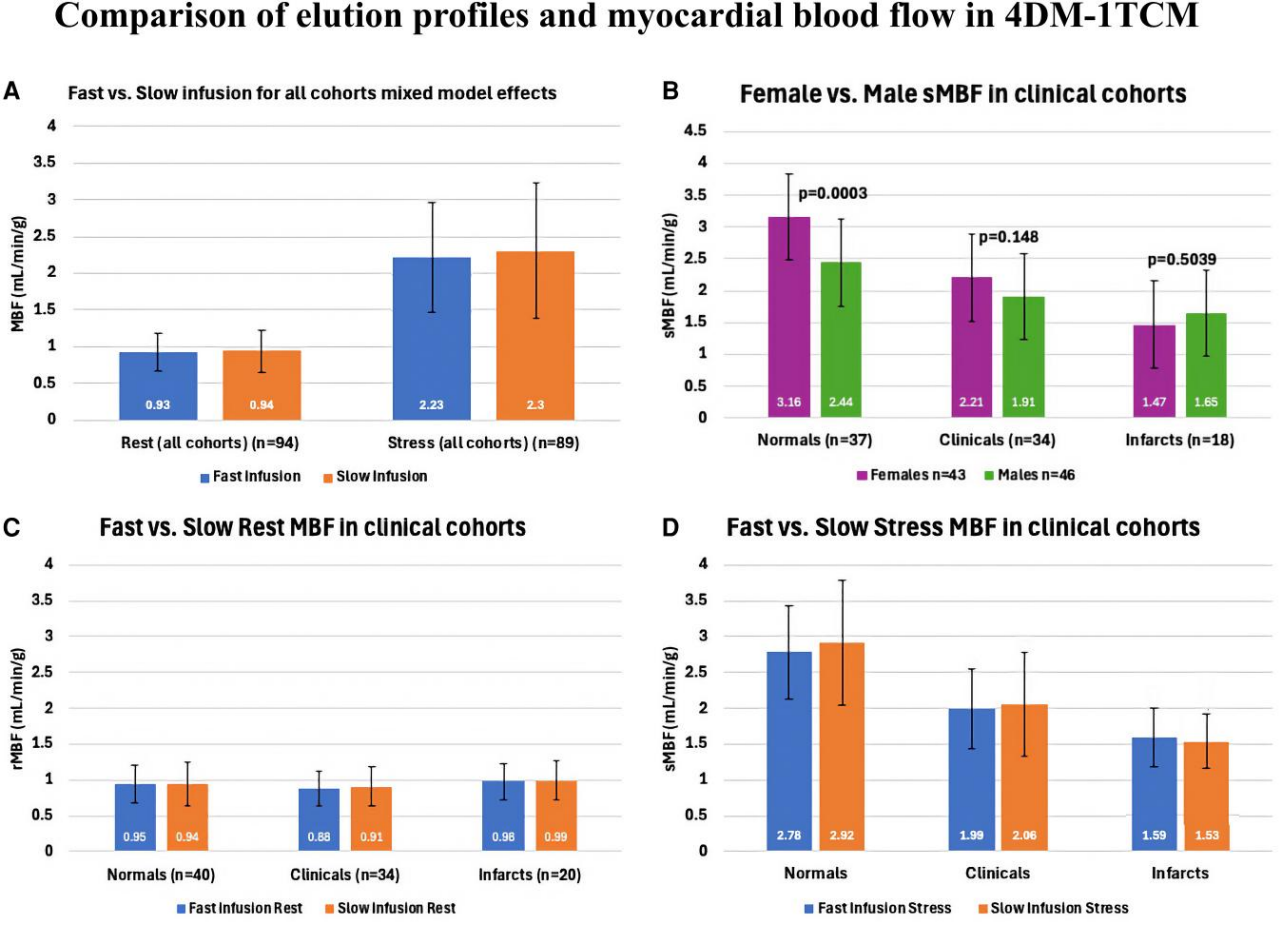
Comparison of elution profiles and myocardial blood flow.

Sensitivity and linear regression analysis demonstrated that there was no test-retest difference as a function of the age of the generator (expressed in weeks or days), which is demonstrated in Supplementary data online, *Table S4* and Supplementary data online, *Figures S5* and *S6*. As demonstrated in *Figure 3* and *Table 2*, in all patients, there was no difference in rMBF and sMBF between F and S profiles (rMBF 0.93 ± 0.25 vs. 0.94 ± 0.29 mL/min/g, *P* = 0.727; sMBF 2.23 ± 0.75 vs. 2.30 ± 0.92 mL/min/g, *P* = 0.082).

**Table 2:** Fast vs. slow infusion MBF by cohort via a multifactor ANOVA mixed model for the crossover design. Observed mean (standard deviation) with corresponding ***P*-value from mixed model**

	Fast	Slow	*P*-value
Rest (all cohorts) (mL/min/g) (n = 94)	0.93 (0.254)	0.94 (0.288)	0.7267
Stress (all cohorts) (mL/min/g) (n = 89)	2.23 (0.749)	2.30 (0.917)	0.0816

The secondary analysis using a multifactor ANOVA mixed model for the cross-over design considering possible modifying effects of Clinical Group and Sex is demonstrated in *Tables 3A&B*. It did not show any significant interaction with either Rest or Stress. For mean rMBF there was no significant interaction by infusion group. For sMBF, only Group by Sex was significant (Females sMBF 3.16 and Males sMBF 2.44, *P* = 0.0003).

**Table 3:** Resting and stress MBF by infusion group. Values are observed means (standard deviation); ***P*-values are from mixed models comparing Fast vs. Slow within each subject group. For rest, no interaction was significant. For stress only Group by Sex was significant (for Fast vs. Slow)**

Whole Heart Rest MBF (mL/min/g)	All subjects (n = 95)	Fast (n = 47)	Slow (n = 48)	*P*-value (F vs. S)
Normals (n = 40)	0.94 (0.287) (80)	0.95 (0.265) (n = 40)	0.94 (0.310) (n = 40)	NA*
Clinicals (n = 34)	0.89 (0.256) (68)	0.88 (0.239) (n = 34)	0.91 (0.274) (n = 34)	NA*
Infarcts (n = 20)	0.98 (0.259) (40)	0.98 (0.252) (n = 20)	0.99 (0.272) (n = 20)	NA*

Whole Heart Stress MBF (mL/min/g)	All subjects (n = 89)	Fast (n = 47)	Slow (n = 48)	*P*-value (F vs. S)
Normals (n = 37)	2.85 (0.768) (74)	2.78 (0.654) (n = 37)	2.92 (0.871) (n = 37)	NA*
Clinicals (n = 34)	2.02 (0.644) (68)	1.99 (0.560) (n = 34)	2.06 (0.725) (n = 34)	NA*
Infarcts (n = 18)	1.56 (0.392) (38)	1.59 (0.416) (n = 19)	1.53 (0.376) (n = 19)	NA*

Stress MBF (mL/min/g)	All subjects (n = 89)	Females N = 43	Males N = 46	*P*-value (F vs. S)
Normals (N) (n = 37)	2.85 (0.768) (74)	3.16 (0.683) (n = 42)	2.44 (0.683) (n = 32)	0.0003
Clinicals (C) (n = 34)	2.02 (0.644) (68)	2.21 (0.720) (n = 26)	1.91 (0.571) (n = 42)	0.1479
Infarcts (I) (n = 18)	1.56 (0.392) (36)	1.47 (0.359) (n = 18)	1.65 (0.415) (n = 18)	0.5039

^*^ *P*-values not reported as there was no interaction in the mixed model analysis. The observed means (standard deviations) are reported for reference only.

### Comparison of precision between 1-TCM-4DM vs. GSRM-HeartSee across all cohorts

For resting F-F profiles and stress F-S profiles, 1TCM-4DM and GSRM-HeartSee demonstrated similar precision across all patient populations (COV rest F-F 12.7% vs. 11.5%, RC% 24.2% vs. 21.5%—1TCM-4DM vs. GSRM-Heartsee, COV stress F-S 9.6% vs. 9.6%, all *P* values >0.05). However, rMBF precision with the Slow profile within 1TCM-4DM was significantly worse compared to GRSM-HeartSee (COV 17.4% vs. 11.3%, *P* = 0.011, RC% 32.9% vs. 22.6%).

### Precision of resting MBF in 1-TCM-4DM vs. GSRM-HeartSee in regions of TMS

Precision metrics favoured GSRM-HeartSee, which showed markedly lower coefficients of variation and repeatability coefficients compared to 1-TCM-4DM (COV% Fast: 8.1 vs. 21.2; Slow: 8.0 vs. 25.1; both *P* < 0.001).

### Accuracy of rMBF in 1-TCM-4DM vs. GSRM-HeartSee in regions of TMS

**Figure 4:**
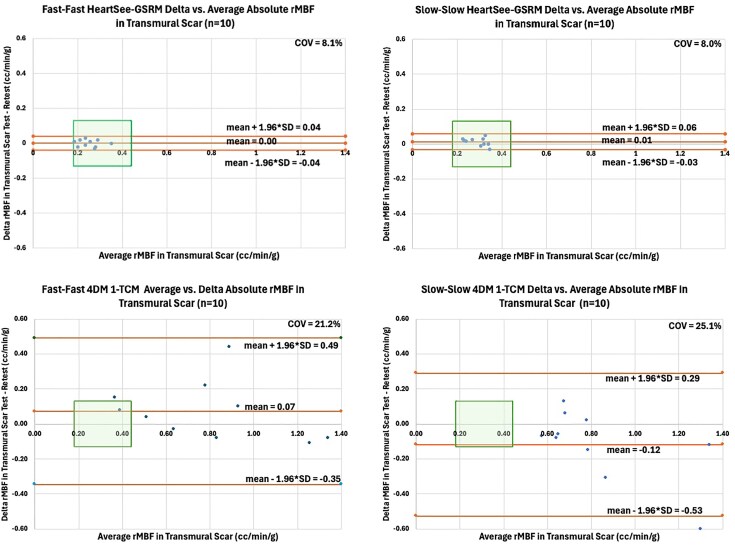
Modified Bland-Altman plots comparing rMBF test–retest agreement across kinetic models and infusion protocols. Each panel shows absolute rMBF values for F–F and S–S infusions using GSRM and 1-TCM (4DM). The green box denotes the physiologically expected range for rMBF in transmural scar (0.18–0.44 mL/min/g) and the repeatability limits corresponding to a 12.7% COV, derived from a weighted average of prior Rb-82 studies (*Table 4*). GSRM results stay within these bounds across protocols, whereas 1-TCM frequently exceeds them.

In transmural scar segments, where true resting myocardial blood flow (rMBF) is expected to average approximately 0.30 mL/min/g and not exceed 0.44 mL/min/g, GSRM produced values consistent with this physiological reference under both infusion profiles (Fast: 0.25 ± 0.05; Slow: 0.29 ± 0.05 mL/min/g), with no significant difference between Fast and Slow conditions (*P* = 0.08). In contrast, 1-TCM substantially overestimated rMBF under both infusion profiles (Fast: 0.79 ± 0.33; Slow: 0.82 ± 0.27 mL/min/g, *P* = 0.791), again with no significant difference between profiles. However, for both Fast and Slow infusions, rMBF estimates were significantly higher with 1-TCM-4DM compared to GSRM-HeartSee (*P* < 0.001). Per cent radiotracer uptake (%RU) was similar across models and infusion conditions (*P* > 0.75). Comparison of precision and accuracy between models are demonstrated in *Figure 4*.

## Discussion

This study evaluated the impact of F (50 mL/min) vs. S (20 mL/min) Rb-82 infusion profiles on quantitative myocardial perfusion metrics using a 1-TCM-4DM. While our prior study examined precision of quantitative perfusion using GSRM-HeartSee, this study is the first to systematically compare precision between F and S infusion profiles using the most widely adopted 1-TCM implementation in 4DM software. For Objective 1, our current study found better precision in 1-TCM utilized by 4DM with a fast infusion of Rb-82. For Objective 2, using the same datasets and protocol for all patient cohorts, precision was similar between 1TCM-4DM and GRSM-HeartSee provided a fast infusion was used. However, precision worsened with a slow infusion using 1TCM-4DM. For Objective 3 (accuracy and precision of rMBF within transmural scar), GSRM-HeartSee demonstrated superior precision and accuracy when compared to 1TCM-4DM. 1-TCM-4DM consistently overestimated rMBF in TMS regardless of infusion rate.

### Comparison to prior studies

There are numerous studies seeking to quantify same-day and different-day precision of MBF metrics. These are summarized in *Table 4*. The key studies using Rb-82 infusions that merit mention are Klein et al., Bober et al., Kitkungvan et al., and Choueiry et al.^12–14,26^ Klein and Choueiry both tested a generator system with two distinct Rb-82 infusion profiles: constant flow (CF) profile and constant activity (CA) profile. The CF profile infuses at a rate ranging from 15–30 mL/min. The CA profile automatically adjusts the flow rate to elute constant activity (CA) throughout the life of the generator. Klein et al. studied 22 patients and reported nonparametric RC = 21% using CA-CA infusions and a 1-TCM (FlowQuant). They also reported a nonparametric RC = 36% comparing CA-CF infusion with their 1-TCM. However, there were 3 outliers in that study which were very likely erroneous, given that resting MBF >3.0 mL/min/g is not physiologically plausible. Furthermore, the definition of RC requires identical conditions which were not present in the CA-CF population. Thus, their statistical methods for this comparison may have been flawed. In addition, they did not test constant flow vs. constant flow, nor did they test a fast infusion profile at 50 mL/min.

**Table 4:** Literature review of precision of resting myocardial blood flow using various techniques

Author	Technique/ tracer	Software/ model	Protocol	COV	RC% (abs)	Year
Nagamachi^17^ n = 30	PET N13	2-CM	Same day	15%	31% (0.07)	1996
Kaufmann^18^ n = 20	PET O15H2O	1-TCM	Same day	10.9%	22% (0.28)	1999
Wyss^19^ n = 11	PET O15H2O	PMOD 1-TCM	Same day	18.4%	37% (0.58)	2003
Schindler^20^ n = 20	PET N13	2-CM	Same day	20.56%	40.3% (0.26)	2007
			Diff day	21.0%	41% (0.26)	
Manabe^21^ n = 15	PET Rb82	1-TCM	Diff day	16.6%	32.4% (0.21)	2009
Sdringola^22^ n = 107	PET Rb82	GSRM	F-F Diff Day	18%	35% (0.25)	2011
Efseaff^23^ N = 27	PET Rb82	1-TCM	CA-CA Same day	17%	33% (0.26)	2012
Ocneanu^24^ N = 12	PET Rb82	FlowQuant 1-TCM	CA-CA Same day	NR	15.8% (0.21)^a^	2017
Kitkungvan^14^ n = 120	PET Rb82	HeartSee GSRM	F-F Same day	10.7%	21% (0.08)	2017
			Diff day	21.1%	45% (0.17)	
Klein^13^ n = 22	PET Rb82	FlowQuant 1-TCM	CA-CA Same day	NR	21% (0.18)^a^	2018
Otaki et al^25^ n = 21	PET Rb82	Cedars 1-TCM Testing repeatability with MoCo^b^ and AIF^c^ location	F-F Same day	MCLA 8.8%	17.2% (0.16)	2019
				MCLV 9.2%	18.0% (0.18)	
				NoMC 16.0%	31.4% (0.32)	
Choueiry^26^ n = 140	PET Rb82	4DM 1-TCM	CA-CA Same day	16%	31% (0.25)	2022
Bober et al^12^ n = 94	PET Rb82	HeartSee GSRM	F-F Same day	11.5%	21.5% (0.16)	2024
			S-S Same day	11.3%	22.6% (0.18)	
Koos et al N = 94	PET Rb82	4DM 1-TCM	F-F Same day	12.9%	24.2% (0.18)	2024
			S-S Same day	17.4%	32.9% (0.40)	
**Weighted average same day all tracers**				**13**.**1%**	**26.1%** (**0.21)**	
**Weighted average diff day all tracers**				**19**.**2%**	**39.5%** (**0.21)**	
**Weighted average same day Rb-82 ONLY**				**12**.**7%**	**25.4%** (**0.21)**	
**Weighted average diff day Rb-82 ONLY**				**19**.**6%**	**40.3%** (**0.21)**	

^a^Non-parametric statistics were used.

^b^Motion correction.

^c^Arterial input function.

Choueiry et al. studied 140 paired studies using CA-CA infusions and 4DM 1-TCM software.^26^ Their results showed an RC = 31% and COV = 16% closely mirroring our findings using S-S infusions (RC = 32.9% and COV = 17.4%). Importantly, we noted statistically better precision with F-F infusions (RC = 24.2% and COV = 12.9%). It’s important to recognize that the CA profile infusion automatically adjusts flow rates between 10 and 30 mL/min to achieve constant activity and is visually similar to the 20 mL/min slow profile over the life of the generator. Hence, the combination of data from Choueiry et al. and the current study strongly suggest that precision is better with fast infusion (50 mL/min) when using the 1-TCM in 4DM. Data from Kitkungvan et al., Bober et al., and the current study are consistent with same-day precision using a Fast infusion being described as RC = 21–24% and COV = 10–13%.

In the current study, although F–F infusions yielded slightly higher average mean difference (∼10%) compared to S–S (∼2%), care must be taken in interpreting bias as a meaningful measure of precision. In this context, the mean difference is expressed as a percentage, not an absolute value, and the magnitude remains relatively small in clinical terms. In the S–S infusion group, measurement differences were more variable and symmetrically distributed around zero, resulting in a low mean bias. However, this broader variability increased the standard deviation of differences, widened the limits of agreement in the Bland-Altman plot, and led to a higher repeatability coefficient. In contrast, the F–F protocol demonstrated more consistent measurements with fewer extreme values, resulting in a narrower distribution and lower standard deviation. This translated into a lower coefficient of variation (COV). Since COV captures both bias and variability (as the standard deviation of differences normalized to the mean), it offers a more complete measure of precision. Therefore, despite a modest increase in bias, the F–F infusion protocol demonstrated superior repeatability and overall precision compared to S–S. Taken together, these findings support the conclusion that F–F infusions provide superior overall precision in MBF quantification using the 1-TCM.

### Accuracy of rMBF in transmural scar regions

Accuracy was assessed in regions of transmural scar, where true resting myocardial blood flow (rMBF) is expected to fall between 0.18 and 0.44 mL/min/g.^27^  *Figure 4* displays a series of modified Bland-Altman plots using absolute rMBF values, comparing test–retest differences across models (GSRM vs. 1-TCM) and infusion protocols (Fast–Fast vs. Slow–Slow). The green box in each plot denotes the expected range for both accurate rMBF and acceptable precision, defined by the 12.7% COV established from a weighted average of same-day Rb-82 studies (*Table 5*).

**Table 5:** Summary of rMBF and precision in transmural scar segments

		1-TCM	GSRM	*P*-value
rMBF	F	0.79+/−0.33	0.25+/−0.05	*P* < 0.001
	S	0.82+/−0.27	0.29+/−0.05	*P* < 0.001
%RU	F	45.3+/−3.4	45.6+/−3.3	*P* = 0.849
	S	45.4+/−3.9	45.8+/−4.5	*P* = 0.756
COV%	F	21.2	8.1	*P* < 0.001
	S	25.1	8.0	*P* < 0.001
RC%	F	41.8	16.4	NA
	S	36.3	15.2	NA

GSRM demonstrated both high accuracy and precision under both infusion protocols. All paired rMBF measurements fell within the green box on the Bland-Altman plots—indicating that values were within the physiologically expected range (0.18–0.44 mL/min/g) and within the limits of repeatability defined by a 12.7% COV. Both F–F and S-S GSRM achieved a low COV at ∼8.0%.

In contrast, 1-TCM results consistently fell outside the green box on the Bland-Altman plots in terms of both accuracy and precision. Both S–S and F–F infusions using 1-TCM produced rMBF values that repeatedly exceeded the expected upper limit of 0.44 mL/min/g in transmural scar, indicating overestimation in low-flow territories. The observed overestimation of rMBF by the 4DM implementation of the 1-TCM in infarcted territories is likely multifactorial. First, inaccuracies in the arterial input function due to ROI placement or unrecognized motion can disproportionately affect flow estimates in low-perfusion segments. Second, inaccurate LV myocardial boundary segmentation in regions of infarct could cause spill-over effect and erroneously increase rMBF estimates. Third, inaccuracies in partial volume correction factors, especially in thinned myocardium, can lead to overcompensation and inflated MBF values. Fourth, if extraction fraction corrections are not accurately modelled at low flow states, the kinetic model may default to erroneously high values. Potential corrective strategies could include improved contour propagation algorithms with infarct-specific territories, direct camera specific PV loss measurements with appropriate adjustments within the model, or implementation of model constraints that limit the physiological range of rMBF in infarcted tissue.

**Figure 5:**
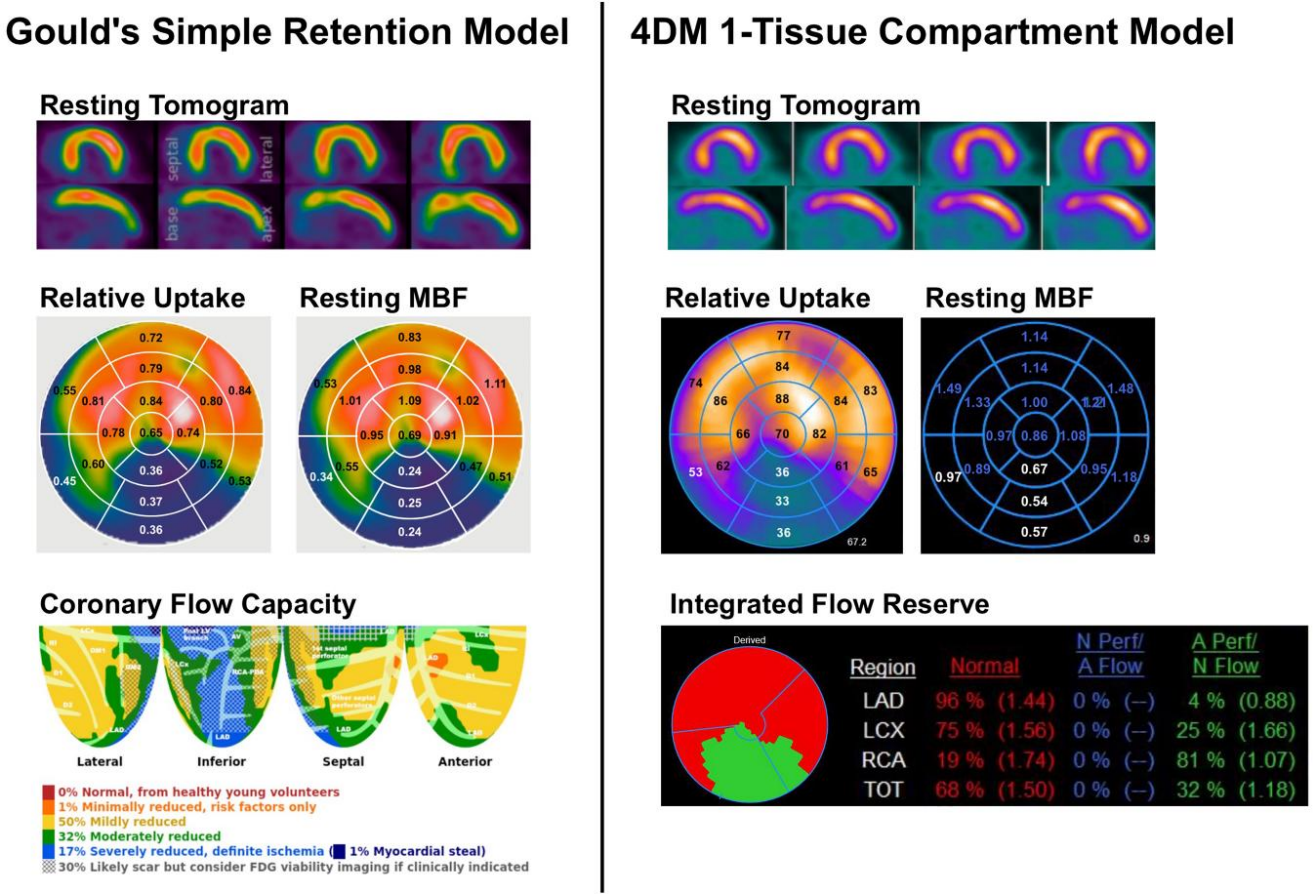
Representative case of a transmural infarct in GSRM and 1-TCM. Representative case of a large, well-demarcated transmural infarct in the inferior wall with minimal tracer uptake. GSRM estimated an average rMBF of 0.27 mL/min/g, consistent with scar tissue, whereas 1-TCM in 4DM overestimated rMBF at 0.69 mL/min/g, despite clear infarction, highlighting 1-TCM’s limitations in low-flow regions. The bottom row shows that GSRM's Coronary Flow Capacity (CFC) correctly identifies severely reduced flow, while 1-TCM’s Integrated Flow Reserve (IFR) misclassifies the infarcted zone as ‘normal.’ This underscores how model differences can lead to clinically significant misinterpretation, particularly in scarred myocardium.

Moreover, the upper and lower bounds of repeatability extended well beyond the limits defined by the 12.7% COV, highlighting degraded test–retest precision. While F–F infusion yielded modestly improved precision compared to S–S (COV = 21.2% vs. 25.1%), both were substantially less precise than GSRM (COV 8%). Further underscoring this limitation, rMBF values derived from 4DM failed to distinguish between healthy volunteers and patients with large transmural infarcts, yielding nearly identical resting flow estimates (Normals: 0.94 vs. Infarcts: 0.98 mL/min/g, *P* = NS; *Table 3*). A representative case illustrating these findings is shown in *Figure 5*.

Importantly, the clinical implications of these findings are not symmetric across the MBF spectrum. In individuals with normal perfusion (e.g. MBF of 2.5–3.5 mL/min/g), moderate inaccuracies or variability have minimal diagnostic consequence. However, in patients with low-flow states, even modest proportional errors can lead to misclassification of disease severity, potentially affecting diagnostic interpretation and clinical decision-making.

GSRM-HeartSee maintained high precision and accuracy in these low-flow conditions whereas 1-TCM-4DM performance deteriorated, showing both increased variability and overestimation of flow. The inaccuracy observed with 1-TCM is most likely attributable to the specific implementation within the 4DM software, rather than the compartmental model itself. Since rMBF is calculated as myocardial uptake divided by the arterial input function—and modified by coefficients such as tracer extraction fraction and partial volume correction factors—inaccuracies in any of these elements can lead to substantial errors in the final rMBF estimate. In this study, relative perfusion images (i.e. myocardial uptake) were nearly identical between GSRM-HeartSee and 1-TCM-4DM, suggesting that the discrepancy stems from errors in the aforementioned components, and possibly from boundary selection as well.

### Generator age

One limitation of our previous work was that MBF estimates were significantly higher in the slow vs. fast infusion group, when using GSRM. First, in sensitivity analyses, both our previous study and the current study demonstrate no impact of generator age with flow values derived using softwares that employ either GSRM-HeartSee or 1-TCM-4DM. Second, GSRM-HeartSee demonstrated slightly higher rMBF (0.69 vs. 0.79) and sMBF (1.94 vs. 2.01) with slow infusion, but there was no difference with 4DM-1-TCM between fast and slow infusions: rMBF (0.93 vs. 0.94, *P* = 0.7267) and sMBF (2.23 vs. 2.3, *P* = 0.082). Third, using GSRM-HeartSee, a delay of infusion would dilute the concentration of radiotracer of the early image which is used to determine input function. A diluted input function would increase calculated myocardial blood flow values. Contrary to GSRM-HeartSee, the arterial input in the 4DM-1-TCM is not impacted by the delay. Therefore, we propose that the difference between the F vs. S in GSRM-HeartSee was not from generator age, but rather from the methodology of the model. Given how the early image is generated using a retention model, one would predict higher flows with slow infusions.

### Software, modelling, and implications

Given the variability in performance observed with 4DM-1-TCM, our findings also suggest that if 1-TCM is used, its clinical utility may be improved by pairing it with a fast infusion protocol, which yielded better precision than the slow protocol in this study. These findings underscore that not only the infusion protocol, but also the modelling approach and software implementation, play critical roles in determining the reliability of quantitative perfusion metrics in clinical practice.

### Limitations

This study evaluated test–retest precision and accuracy of MBF quantification using 1-TCM in 4DM under two Rb-82 infusion profiles, compared with previously published GSRM results in HeartSee. Both models were applied to the same dataset, allowing direct comparison under identical conditions; however, other software platforms or modelling approaches were not assessed, limiting generalizability. Stress repeatability was not tested under matched infusion conditions (F–F or S–S), so RC could not be calculated, and precision at stress was assessed only via COV.

## Conclusions

Using PET-CT assessment of quantitative perfusion, a fast Rb-82 infusion profile (50 mL/min) demonstrated higher test–retest precision than a slow profile (20 mL/min) when using the 4DM software package. With a fast infusion, same-day test–retest precision was comparable between 1TCM-4DM and GSRM-HeartSee, with coefficients of variation (COV) of ∼10–13% across models. In contrast, a slow infusion with 1TCM-4DM yielded reduced precision (COV 17.4%). When applied to identical datasets, 1TCM-4DM consistently overestimated resting MBF in TMS, regardless of infusion rate, and did so with less precision compared to GSRM-HeartSee. In contrast, GSRM-HeartSee demonstrated both accuracy and precision within TMS across F and S infusion profiles.

## Supplementary Material

qyaf132_Supplementary_Data

## Data Availability

The data that supports the findings of this study are available from the corresponding author upon reasonable request.

## Lead author biography

Adrienne Koos is a resident physician of internal medicine at the globally renown Ochsner Clinic Foundation in New Orleans, LA, USA. She completed her undergraduate studies at Western Oregon University and her medical school training at Western University of Health Sciences. Dr Koos has quickly become prolific in her research interests and endeavours despite her short time in training, undertaking research in topics such as PET-CT myocardial perfusion imaging, cardio-oncology, oestrogen effects on cardiovascular health, and the Obesity Paradox.
