# Supplementary material for: The impact of fast vs. slow rubidium-82 infusion profile on precision and accuracy of PET myocardial blood flow perfusion metrics using a 1-tissue compartment model
Source: Eur Heart J Imaging Methods Pract. 2025 Oct 24;3(4):qyaf132. doi: 10.1093/ehjimp/qyaf132 (PMC12602862; doi:10.1093/ehjimp/qyaf132)
Supplement: qyaf132_Supplementary_Data [file qyaf132_supplementary_data.docx]

Supplemental Data

Supplemental Figure 1 and 2. Verification of normality of distributions


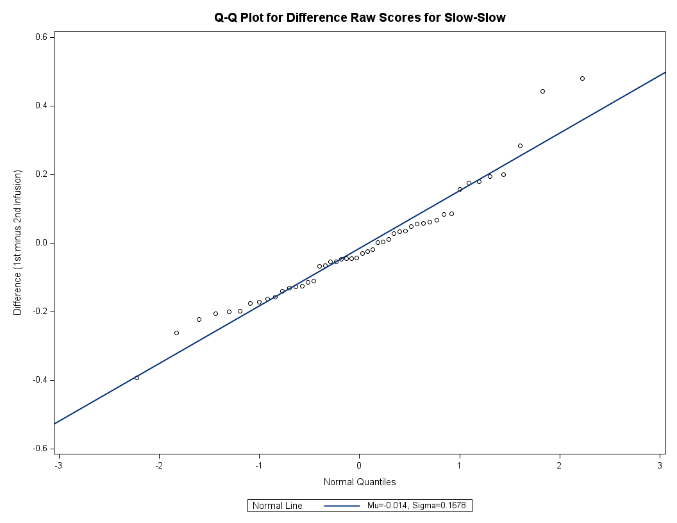

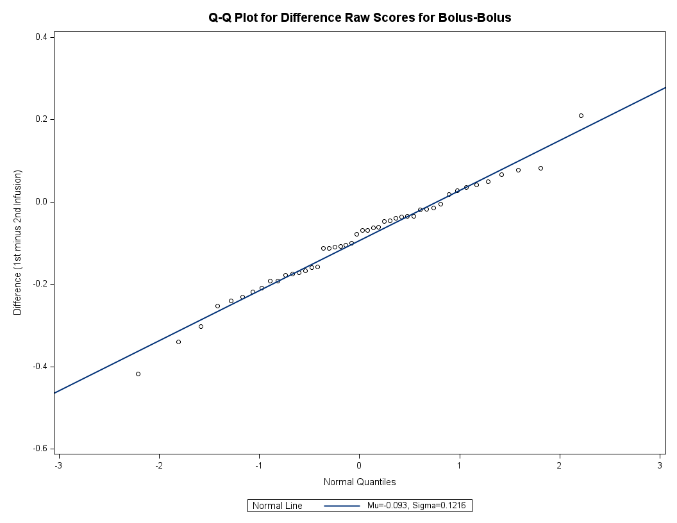


Supplemental Figure 3. Hemodynamic and delta myocardial blood flow during first and second rest and stress scans


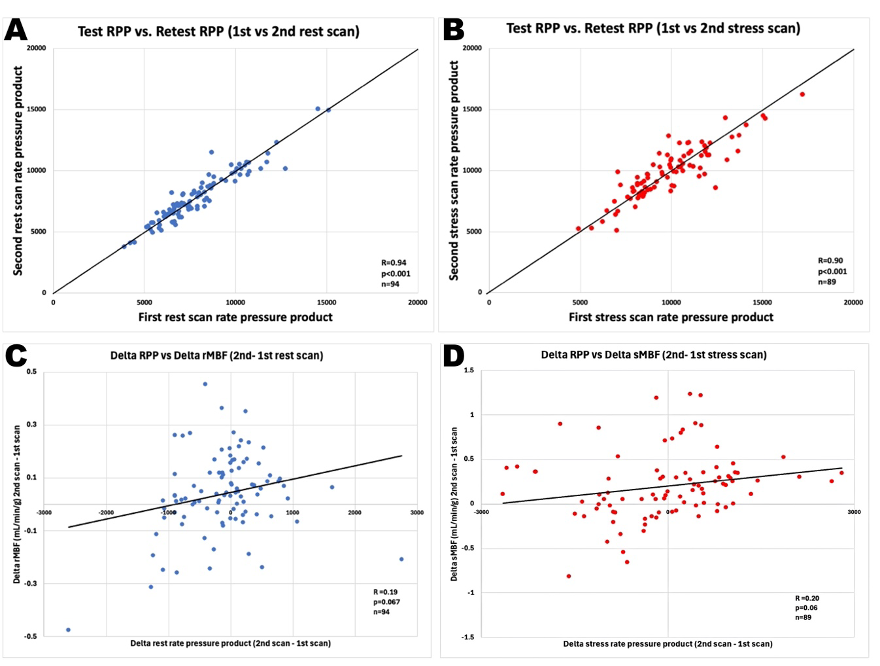


A and B) Excellent correlation between 1^st^ and 2^nd^ rate pressure product (RPP)

C and D) no significant correlation of test-retest changes in myocardial blood flow vs. changes in RPP

Supplemental Figure 4. Bland-Altman plots of absolute values.


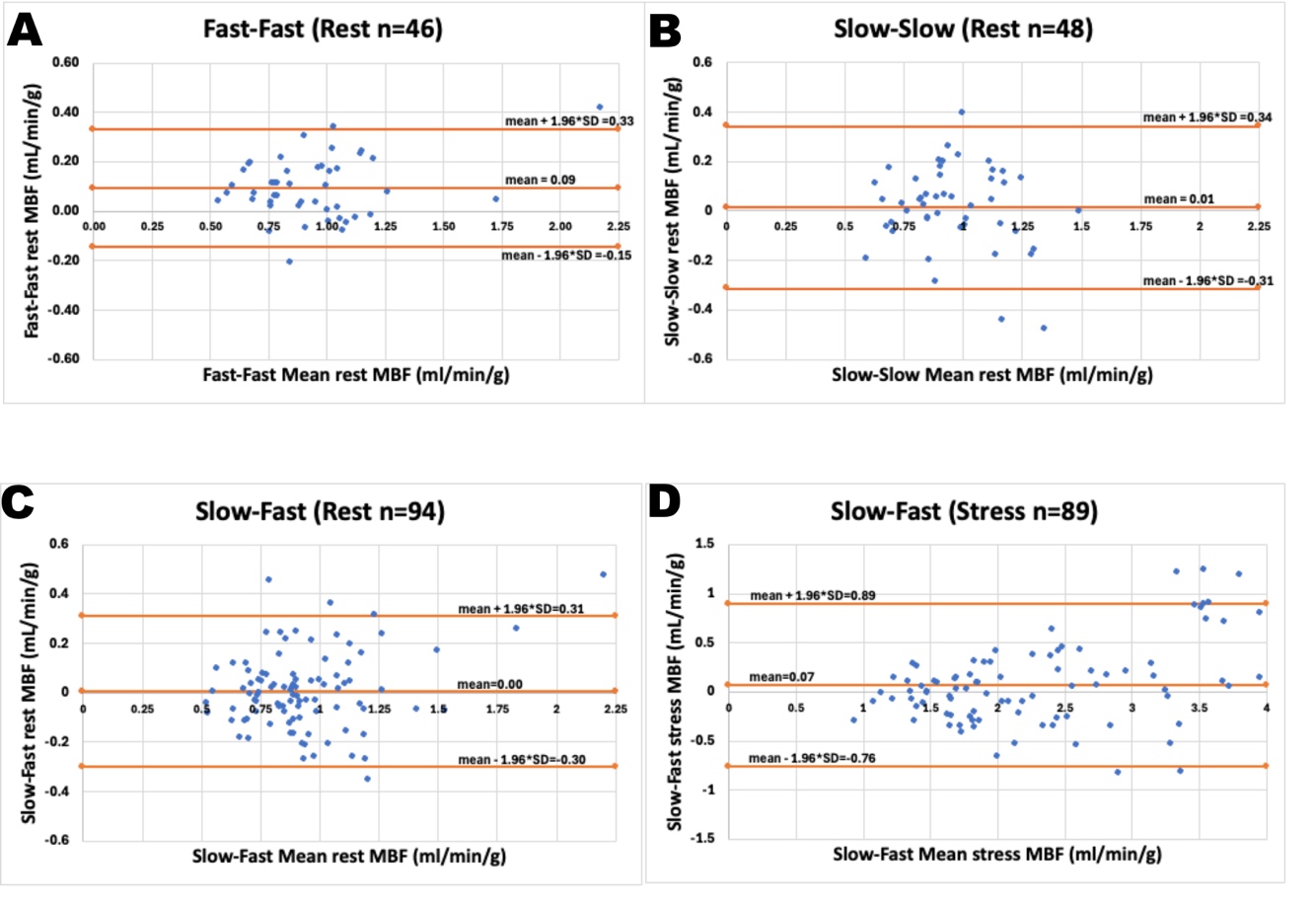


Supplemental Figure 5. Age of generator (as continuous covariate) in the model to estimate IIC demonstrates that there is no impact in test-retest ICC (no trend in rightmost plots).

~~
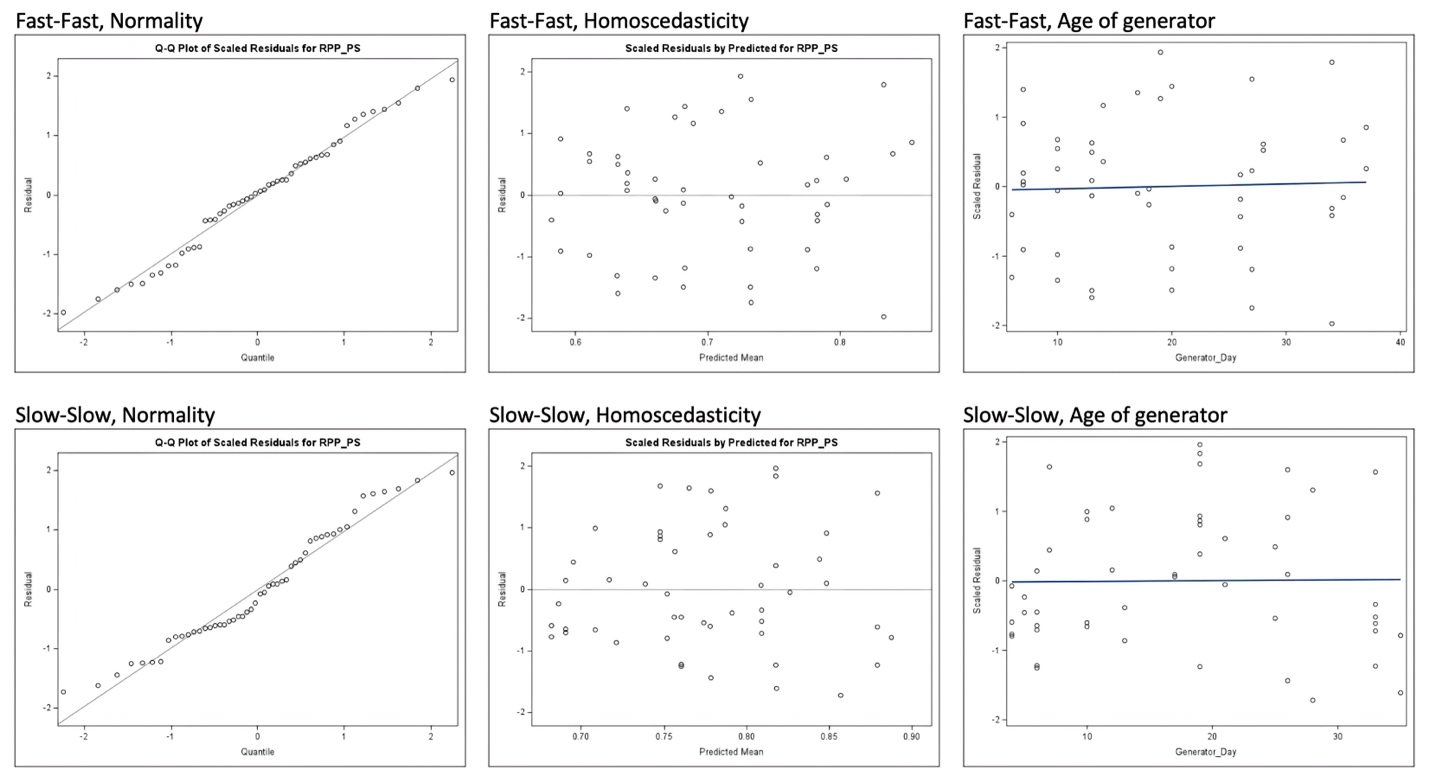
~~

Supplemental Figure 6. Rest and stress myocardial blood flow as a function of generator age in days.


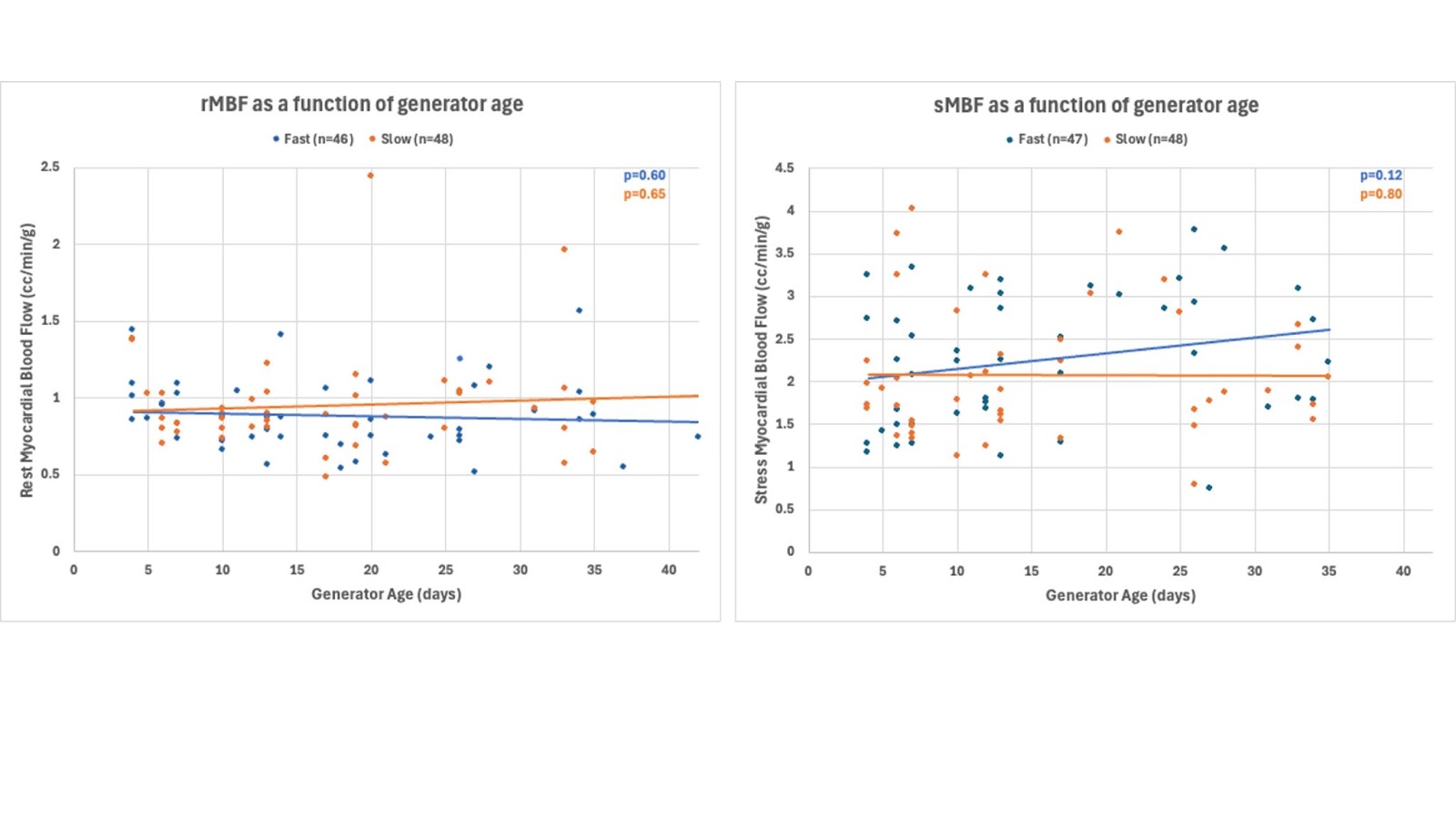


Supplemental Table 1. Verification of normality of distributions

|  | Slow-Slow | Fast-Fast |
| --- | --- | --- |
| N | 48 | 46 |
| Skewness | 0.78 | -0.27 |
| Kurtosis | 1.45 | 0.45 |
| Mean of averages (std dev differences) | -0.014 (0.168) | -0.093 (0.1216) |
| Coefficient of Variation (p=0.13) | -1160.8 | -131.1 |
| Repeatability coefficient (p=0.77) | 1.96*0.168=  0.3293 | 1.96 *0.1216=  0.2383 |
| Shapiro-Wilk | 0.06 | 0.87 |
| Kolmogorov-Smirnov | >0.15 | >0.15 |
| Jarque-Bera | 0.01 | 0.63 |

Supplemental Table 2. Demographic data of participants.

|  | Total | Normals | Clinicals | Infarcts | p-value |
| --- | --- | --- | --- | --- | --- |
| Number of participants | 94 | 40 | 34 | 20 |  |
| Female Sex | 47(49%) | 24(60%) | 13(39%) | 10(50%) |  |
| Age (mean±SD) years | 45.2±17.6 | 30.6±5.2 | 53.3±17.2 | 61.3±10.7 | <0.001 |
| BMI (mean± SD) m^2^/kg | 26.6±6.0 | 22.9±2.5 | 28.0±5.6 | 31.7±7 | <0.001 |
| Diabetes | 18 (19%) | 0 | 6(18%) | 12(60%) | <0.001 |
| Hypertension | 35 (37%) | 0 | 18(55%) | 17(85%) | <0.001 |
| Hyperlipidemia | 37 (39%) | 0 | 21(64%) | 16(80%) | <0.001 |
| Prior PCI | 26 (27%) | 0 | 9(27%) | 17(85%) | <0.001 |
| Prior CABG | 8 (8%) | 0 | 2(6.1%) | 6(30%) | <0.001 |
| LV ejection fraction (rest) | 55±11.7 | 59.8±6 | 59.9±8 | 37.1±8 | <0.001 |

*Note.* Reprinted from Bober et al, 2024, J Nucl Cardiol.

Supplemental Table 3. Hemodynamic data of subjects. Repeated measures ANOVA with interaction between time by Sex. If interaction was not significant then p-values for main effects Period and Sex are reported. Notable comparisons are times that may show a difference in means after Tukey adjustment.

| Parameter | Rest (N=94) | | | | |
| --- | --- | --- | --- | --- | --- |
|  | Period 1, Rest  (Scan #1) | Period 2, Rest  (Scan #2) | Period 3, Rest  (Scan #3) | Time | Notable Time comparisons |
| SBP (mmHg) | 119.1 (23.3) | 118.0 (24.4) | 116.9 (23.1) | 0.0575 | R1 v. R3 p=0.044 |
| DBP (mmHg) | 64.9 (15.0) | 62.5 (14.8) | 62.1 (13.7) | 0.0016 | R1 v. R2 P=0.0117; R1 v. R3 p=0.0025 |
| HR (bpm) | 66.3 (10.6) | 66.0 (10.6) | 67.2 (10.9) | 0.0211 | R2 v. R3 P=0.0218 |
| RPP (bpm x mmHg) | 7916 (2111) | 7804 (2099) | 7865 (2040) | 0.2743 | na |
|  |  |  |  |  |  |
|  | Stress (N=94) | | |  |  |
|  | Period 1, Stress  (Scan #1) | Period 2, Stress  (Scan #2) | Time |  |  |
| SBP (mmHg) | 114.9 (23.1) | 114.9 (29.7) | 0.9955 |  |  |
| DBP (mmHg) | 58.1 (14.1) | 57.6 (12.6) | 0.4895 |  |  |
| HR (bpm) | 85.6 (16.5) | 84.1 (12.7) | 0.1442 |  |  |
| RPP (bpm x mmHg) | 9787 (2602) | 9644 (2888) | 0.5840 |  |  |

SBP systolic blood pressure, DBP diastolic blood pressure, HR heart rate, RPP rate pressure product (HR x SBP), bpm beats per minute, mmHg millimeters of mercury, period 1^st^, 2^nd,^ or 3^rd^ infusion

Supplemental Table 4. ICCs were computed for Supplemental Figure 3 for groups by Age of Generator with results shown in the Table below. ICC remains unchanged over age of generator for test-retest settings.

| Figure | All | | | Generator Age 1-3wks | | Generator Age 4-6wks | |
| --- | --- | --- | --- | --- | --- | --- | --- |
|  | N | ICC | Generator Age in days (p-value) | N | ICC | N | ICC |
| Rest F-F (3A) | 46 | 0.8454 | 0.2433 | 33 | 0.7981 | 9 | 0.8720 |
| Rest S-S (3B) | 48 | 0.7243 | 0.7639 | 34 | 0.6159 | 8 | 0.7570 |
| Rest S-F (3C) | 94 | 0.8446 | NA | 67 | 0.8249 | 27 | 0.8805 |
| Stress F-S (3D) | 89 | 0.9000 | NA | 66 | 0.9261 | 23 | 0.8445 |
